# Spatiotemporal Distribution, Sources, and Photobleaching Imprint of Dissolved Organic Matter in the Yangtze Estuary and Its Adjacent Sea Using Fluorescence and Parallel Factor Analysis

**DOI:** 10.1371/journal.pone.0130852

**Published:** 2015-06-24

**Authors:** Penghui Li, Ling Chen, Wen Zhang, Qinghui Huang

**Affiliations:** 1 Key Laboratory of Yangtze River Water Environment of the Ministry of Education, College of Environmental Science and Engineering, Tongji University, Shanghai, China; 2 State Key Laboratory of Pollution Control and Resources Reuse, College of Environmental Science and Engineering, Tongji University, Shanghai, China; 3 John A. Reif, Jr. Department of Civil and Environmental Engineering, New Jersey Institute of Technology, Newark, New Jersey, United States of America; Old Dominion University, UNITED STATES

## Abstract

To investigate the seasonal and interannual dynamics of dissolved organic matter (DOM) in the Yangtze Estuary, surface and bottom water samples in the Yangtze Estuary and its adjacent sea were collected and characterized using fluorescence excitation-emission matrices (EEMs) and parallel factor analysis (PARAFAC) in both dry and wet seasons in 2012 and 2013. Two protein-like components and three humic-like components were identified. Three humic-like components decreased linearly with increasing salinity (*r*>0.90, *p*<0.001), suggesting their distribution could primarily be controlled by physical mixing. By contrast, two protein-like components fell below the theoretical mixing line, largely due to microbial degradation and removal during mixing. Higher concentrations of humic-like components found in 2012 could be attributed to higher freshwater discharge relative to 2013. There was a lack of systematic patterns for three humic-like components between seasons and years, probably due to variations of other factors such as sources and characteristics. Highest concentrations of fluorescent components, observed in estuarine turbidity maximum (ETM) region, could be attributed to sediment resuspension and subsequent release of DOM, supported by higher concentrations of fluorescent components in bottom water than in surface water at two stations where sediments probably resuspended. Meanwhile, photobleaching could be reflected from the changes in the ratios between fluorescence intensity (F_max_) of humic-like components and chromophoric DOM (CDOM) absorption coefficient (a355) along the salinity gradient. This study demonstrates the abundance and composition of DOM in estuaries are controlled not only by hydrological conditions, but also by its sources, characteristics and related estuarine biogeochemical processes.

## Introduction

Dissolved organic matter (DOM), ubiquitous in aquatic environment, is a complex mixture of organic substances originating from a wide range of allochthonous and autochthonous sources [1]. DOM constitutes the largest reduced carbon reservoir in aquatic ecosystems [2]. Estuaries link the two largest organic carbon reservoirs (the land and the ocean), and discharge DOM and particulate organic matter (POM) through rivers [3]. Biogeochemical processes that occur in estuaries influence the transfer of DOM between these two large pools [3, 4]. In estuaries, allochthonous DOM input or primary production, and removal of DOM due to biodegradation or photobleaching would influence the mixing behavior between freshwater and seawater endmembers [5–9]. Meanwhile, sediment resuspension, flocculation, particle adsorption and POM dissolution occur in the so-called estuarine turbidity maximum (ETM) region of many large estuaries [10–15], influencing the balance between DOM and POM pools [10, 14].

The Yangtze River (also referred as Changjiang River) is the longest river in China and the third longest river in the world and carries an average of about 30000 m^3^/s of water into the East China Sea through the Yangtze Estuary. The Yangtze Estuary is the runoff–dominated and tide-affected lower part of the Yangtze River with an average tidal range of 2.7 m [16]. Due to shallow depth and tidal forces, there is an ETM region in the Yangtze Estuary. Meanwhile, due to substantial export of terrestrial substances (e.g., nutrients) from the Yangtze River runoff and upwelling currents, red tides frequently occur in the Yangtze Estuary and the adjacent East China Sea [17–21], which could contribute to the DOM pool [22, 23]. Moreover, photobleaching, as an important sink of DOM [24, 25], could occur in the plume zone due to the increasing transparency of seawater, though another study did not observe photobleaching [26]. Many estuarine biogeochemical processes occur in the Yangtze Estuary, making it a good site to study the DOM cycling. Therefore, characterization of the DOM sources and fates in the Yangtze Estuary is critical for understanding DOM dynamics and related processes in large estuaries around the world.

In recent years, there have been some studies concerning distribution and fates of DOM in the Yangtze Estuary. DOM at the Yangtze River outlet could vary significantly in abundance and compositions during a daily tidal cycle [5, 27]. The conservative mixing behavior of DOM, indicated by the linear relationship between dissolved organic carbon (DOC) and salinity and between chromophoric DOM (CDOM, quantified by absorption coefficient) and salinity, was observed in the Yangtze Estuary [26, 28], similar to other large estuaries around the world [6, 11, 29–31]. Meanwhile, some possible sources of DOM had been studied in this region: tributary input, release of interstitial water during sediment resuspension, and release from salt marshes [5, 10, 32, 33]. However, the dominant source of DOM remains unclear in this region and the seasonal and interannual variations are still unknown [10]. Moreover, the photobleaching has not been reported in the Yangtze Estuary due to high turbidity and relatively short water retention time [26], despite its great role in transformation and fate of DOM [24, 25, 34]. Therefore, much work is needed to advance our understanding of DOM dynamics, sources and fates in the Yangtze Estuary.

Due to high sensitivity, low cost and fast response, fluorescence spectroscopy has been widely used in characterizing DOM in natural waters [35, 36]. In recent years, parallel factor analysis (PARAFAC), one three-dimension data decomposing tool, was introduced [37] and combined with fluorescence excitation-emission matrices (EEMs) [38, 39] in the applications of DOM characterization in estuarine environments [6, 10, 28, 32, 33, 40].

This study investigated the seasonal and interannual distribution of DOM in the Yangtze Estuary and its adjacent sea based on four cruises using fluorescence EEMs and PARAFAC. The aims of this study are to: (1) characterize the seasonal and interannual variations of DOM in surface and bottom water samples; (2) characterize the mixing behavior of DOM; (3) identify sources of DOM; (4) and track the photobleaching of DOM in the Yangtze Estuary. To the best of our knowledge, this is the first attempt to quantify the interannual variations of fluorescent DOM (FDOM) and study the potential photobleaching of DOM in the Yangtze Estuary, which holds paramount importance in understanding sources and fates of DOM and biogeochemical processes in large estuaries.

## Materials and Methods

### Ethics statement

No specific permits were required for the described field studies. The location studied is not privately-owned or protected in any way, and the field studies did not involve endangered or protected species.

### Study areas and hydrological settings

**Fig 1:**
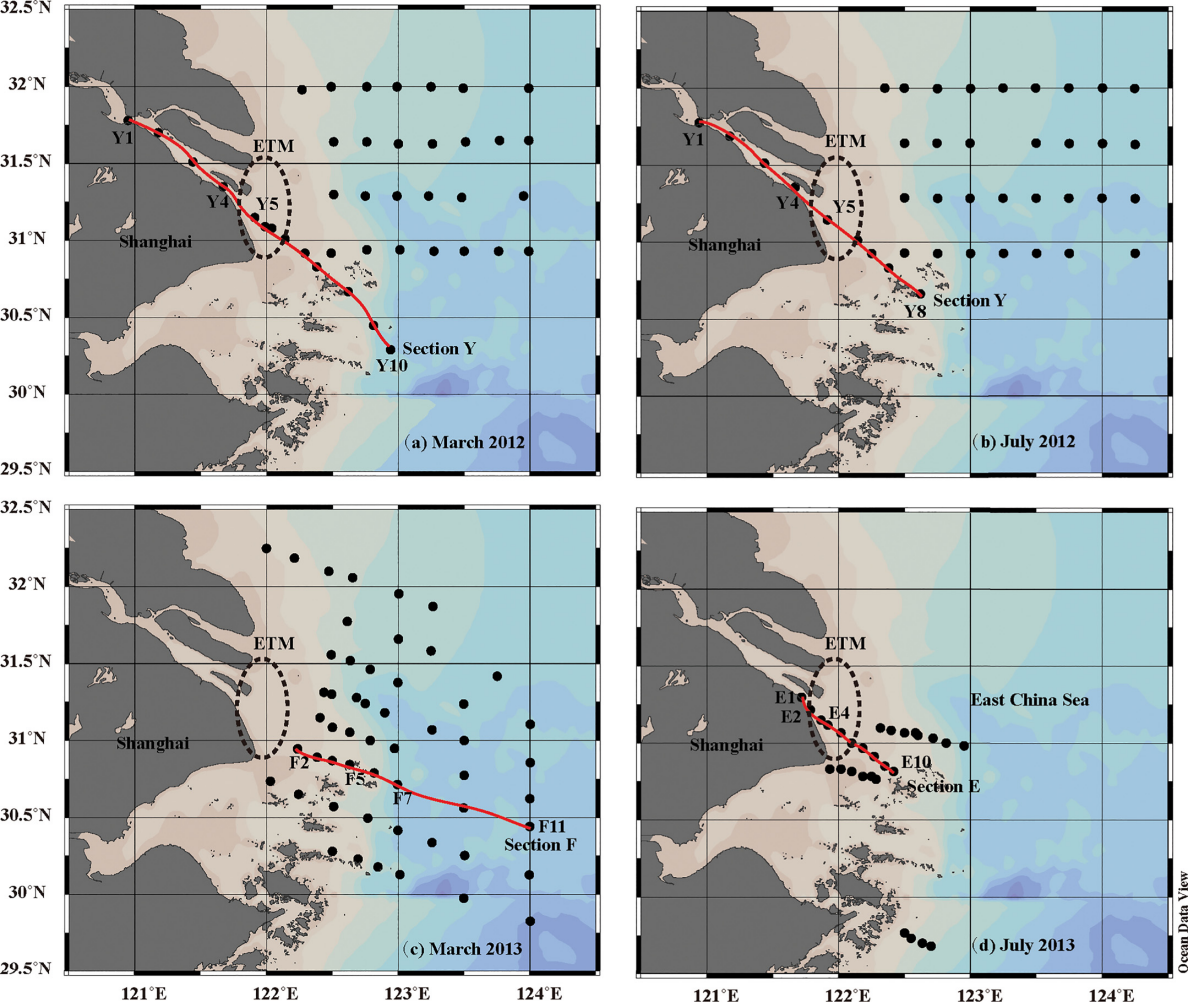
Map of sampling stations in four different cruises. (a) March 2012; (b) July 2012; (c) March 2013; (d) July 2013. Four axial sections of the Yangtze Estuary are connected and marked by red lines. ETM regions are marked by dashed eclipses. Black dots indicate the sampling sites.

Four sampling cruises (Fig 1) were conducted in the Yangtze Estuary and the adjacent East China Sea during dry (March 2012 and 2013) and wet seasons (July 2012 and 2013), respectively. The study areas were located at longitudes between 120.5° E and 124.5° E, and latitudes between 29.5° N and 32.5° N. The lower Yangtze River receives sewage from tributary (namely, the Huangpu River), and salt marshes at Chongming Island and Changxing Island also contribute DOM to the Yangtze River [5]. Saltwater intrusion and sediment resuspension occur in the Yangtze River outlet due to flow variations and tidal forces [10, 14].

The Yangtze River is strongly influenced by East Asian monsoons, showing seasonal and interannual variations of freshwater discharge. Generally, the wet season lasts from May to October, contributing about 70% of the annual discharge; the dry season lasts from November to the next April, contributing about 30% of the annual discharge. The average daily discharge of the Yangtze River is 31832 m^3^/s in 2012 and 26322 m^3^/s in 2013. The average daily discharge in March was 25527 m^3^/s in 2012 and 18116 m^3^/s in 2013, while the value in July was 50881 m^3^/s in 2012 and 41340 m^3^/s in 2013.

### Sample collection and measurement

Sampling campaigns were conducted aboard Patrol Vessels of China Marine Surveillance in 2012 and Marine Science Research Vessels in 2013. Surface water samples were collected with a two-liter stainless steel bucket. Bottom water samples were collected from selected stations along the axial sections of the Yangtze Estuary (e.g., Section F in March 2013 and Section E in July 2013) in Fig 1 using Niskin bottles mounted onto a rosette sampler assembly, equipped with a CTD (Conductivity, Temperature, Depth) recorder (SeaBird, USA). All samples were filtered immediately after they were collected, through precombusted (450°C for 4 h) 0.7 μm glass-fiber membranes (GF/F, Whatman, UK), and then re-filtered using a prewashed 0.22-μm polycarbonate membrane (Millipore, USA). Filtered samples were stored in acid-washed borosilicate glass bottles at -20°C before analysis.

Once samples were taken onboard, temperature (T), salinity (S), dissolved oxygen (DO), and pH of the raw water samples were measured with a portable multi-parameter meter (HQ40d, Hach, USA). Chlorophyll a concentrations (Fl. Chl. a) were determined using a phytoplankton fluorescence analyzer (Phyto-PAM, Walz, Germany).

### Fluorescence and UV-vis spectrum analysis

Fluorescence spectra and intensities were obtained using a fluorescence spectrophotometer (Hitachi F-4500, Tokyo, Japan). Fluorescence intensity (F_max_) was used to indicate the concentration of DOM. EEMs were generated for all samples over excitation wavelengths between 220–400 nm in 3 nm intervals and emission wavelengths between 250–500 nm in 2 nm intervals, with 5 nm bandwidths for both excitation and emission modes. The spectra were recorded at a scan speed of 12000 nm/min, with the voltage of the photomultiplier tube at 700 mV. Milli-Q water (Millipore, USA) was used to check the stability of the instrument daily and to normalize the fluorescence spectra of all samples.

UV-visible absorbance spectrum was measured from 200 nm to 800 nm on a double-beam spectrophotometer (TU-1901, Purkinje General, China) equipped with 5 cm path length quartz cuvettes. Milli-Q water was used as a reference. Average absorbance between 680 nm and 700 nm was used for baseline correction for all samples. The absorption coefficient was converted from absorbance, through multiplying the conversion factor 2.303 and dividing by the path length. In this study, the absorption coefficient at 355 nm (a_355_) was taken as a quantitative measure of CDOM [26, 27, 41]. The ratio of the fluorescence intensity of FDOM components to the absorption coefficient (a_355_) of CDOM, expressed as F_max_:a_355_, was used to track photobleaching [42].

### PARAFAC Modelling

A total of 263 EEMs were collected and analyzed using PARAFAC according to the protocol described by Stedmon and Bro [39]. The PARAFAC analysis was carried out in MATLAB R2013b (Mathworks, USA) with the DOMFluor toolbox (www.models.life.ku.dk). Before PARAFAC modeling, the inner filter effect correction, machine bias, and Raman normalization (hereafter the fluorescence signal reported in Raman Unit, R.U.) were conducted following the procedure reported by Murphy et al. [43]. Moreover, to avoid the influence of low accuracy data due to relatively low lamp output in some lower wavelength regions, the EEMs for PARAFAC modelling only cover the excitation range of 241–400 nm and emission range of 290–550 nm. Also, a non-negativity constraint was applied to the parameters to allow only chemically relevant results. The PARAFAC models with two to seven components were computed for the EEMs. Five components (C1-C5) were determined primarily based on residual analysis, split half analysis, Tucker’s congruence coefficients and visual inspection [39].

## Results

### Characterization of FDOM

**Fig 2:**
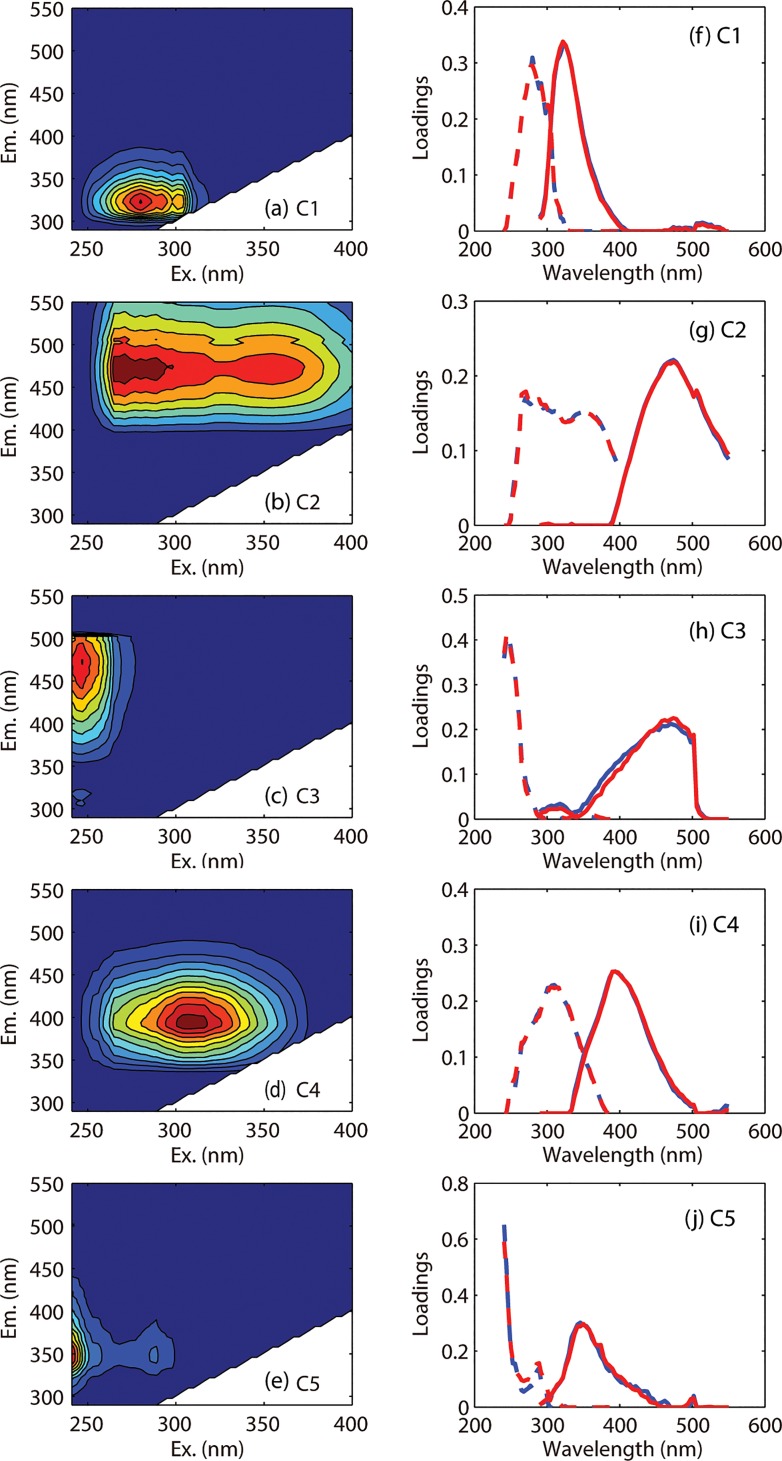
Contour plots and spectral characteristics of five fluorescent components identified by PARAFAC. The line plots present split-half validation data. Excitation (short-dashed lines) and emission (solid lines) loadings are presented for two independent halves of the dataset (red and blue lines).

Five fluorescent components were identified using EEMs-PARAFAC, including two protein-like components (C1 and C5), and three humic-like components (C2, C3 and C4) (Fig 2). Component spectral characteristics and split-half validation data are presented in Fig 2. All components identified in this study have been reported in previous PARAFAC models [33, 40, 44–47] and three components (C1, C2 and C4) have been matched (Similarity Score>0.95) in the OpenFluor database [48]. For instance, both C1 and C5 have been commonly reported as protein-like substances that are sensitive to microbial degradation [36, 46, 49]. C1, resembling the traditionally defined fluorophore B, is tyrosine-like and more degraded peptide material, while C5, resembling the fluorophore T, is tryptophan-like but less degraded peptide material [36, 46]. Querying the OpenFluor database, C1 is found to be matched to a protein-like component observed in prairie lakes (C5 [44]). However, previous studies also show that some non-protein-like substances (e.g., lignin phenols, indoles) could contribute to the protein-like fluorophore T and B [50]. Humic-like C2 and C3 probably originated from allochthonous, terrestrial-derived humic substances that are resistant to microbial degradation [47, 51]. C2, commonly found in both freshwater and seawater [32, 33, 38, 41], resembles a combination of the traditionally defined fluorophore A and C [36], and matches closely to humic-like components reported in the OpenFluor database (C1 [45], C1 [52], C2 [53]). C3, similar to the traditionally defined fluorophore A [36], is associated with C2 [54]. However, the ratio between these two components varied with their sources and environments [13, 36, 54]. C4, with similar characteristics of the fluorophore M, was previously considered as marine humic-like substances [36] or microbial-derived humic-like substances [47], or byproducts of photobleaching of the fluorophore C [25, 55]. Querying the OpenFluor database, C4 is found to be matched to previous humic-like PARAFAC components (C3 [40], C2 [44], C4 [56], C4 [57]).

### Spatiotemporal Distribution of FDOM

#### General description of FDOM distribution

**Fig 3:**
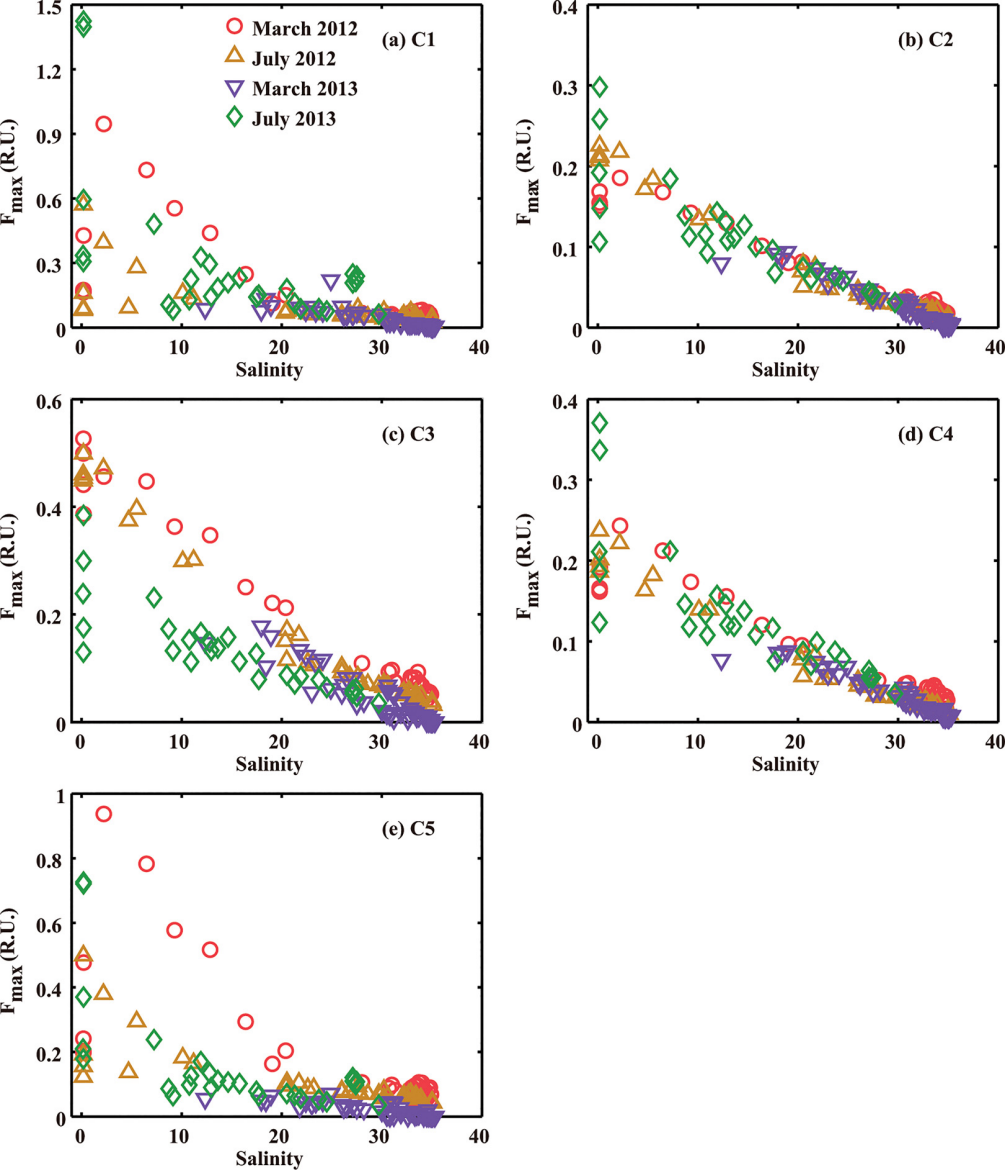
Fluorescence intensity (F_max_) of five fluorescent components (C1-C5) versus salinity.

Fig 3 shows that fluorescence intensity (F_max_) of five fluorescent components (C1-C5) was high in freshwater or low-salinity estuarine water, suggesting that the FDOM distribution was dominated by the freshwater input [58]. All five components decreased with increasing salinity. In particular, two protein-like components (C1 and C5) decreased sharply with increasing salinity in the range of 2 to about 20 and then decreased slowly as the salinity continued to increase, suggesting removal in the salinity range of 2–20. Differently, three humic-like components decreased linearly with increasing salinity, showing dependence on salinity in all four cruises (*r*>0.90, *p*<0.001) as shown in S1 Table, suggesting conservative mixing behaviors.

#### FDOM variations along the axial section of the Yangtze Estuary

**Fig 4:**
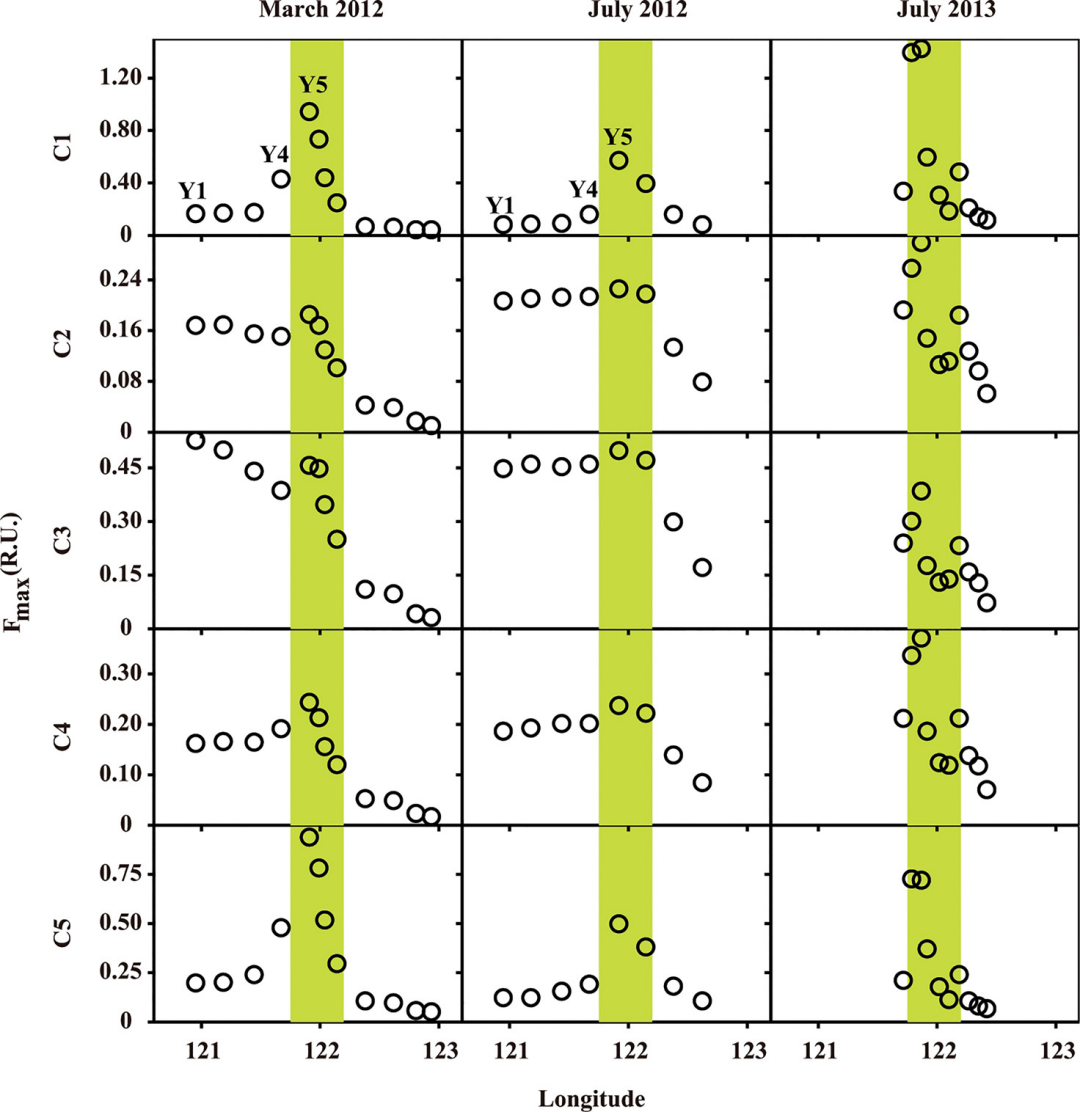
Variations of five fluorescent components in surface water samples along the axial section of the Yangtze Estuary. ETM regions are indicated by green areas.

Along the axial section of the Yangtze Estuary, most FDOM components had minor changes in their concentrations initially (in the Yangtze River), and then increased sharply to the maximum in the ETM region, followed by a rapid decline offshore (Fig 4). Protein-like components (C1 and C5) had much more increases than the humic-like components (C2, C3 and C4), though all five components reached the maximum value in ETM regions. In 2012, C1 had increased by four to five times from station Y3 to station Y5, while C5 increased by two to three times (Fig 4). On the other hand, there were different patterns in 2012 between March and July. The maximum values observed in the ETM region for two protein-like components were lower in July (0.570 R.U. for C1 and 0.499 R.U. for C5) than in March (0.946 R.U. for C1 and 0.937 R.U. for C5) during 2012, suggesting significant variations of protein-like components between seasons. Different from two protein-like components, the concentrations of humic-like components (C2, C3 and C4) were just slightly higher in ETM region in July 2012 than in March 2012, indicative of low sensitivity of humic-like components to the seasonal or freshwater discharge changes.

**Fig 5:**
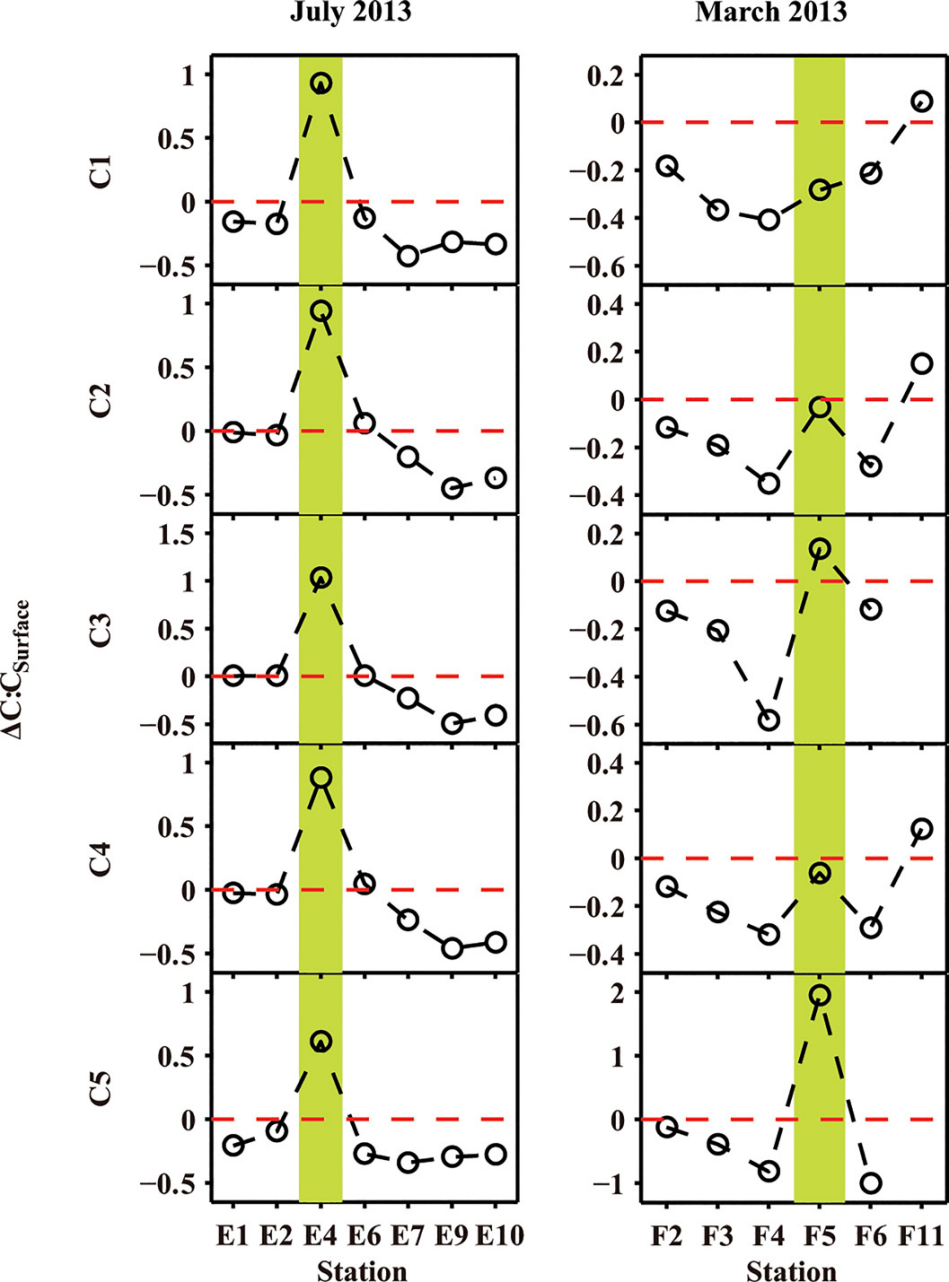
Relative differences between surface and bottom FDOM concentrations along the axial section of the Yangtze Estuary in 2013. The left is for section E in July 2013, and the right is for section F in March 2013. ΔC is calculated as the component concentration in bottom water subtracting the component concentration in surface water. Positive ΔC means a higher component concentration in bottom water than that in surface water.

Differences between the surface and bottom FDOM concentrations could indicate the vertical mixing conditions and the locations of potential FDOM sources [59]. Fig 5 shows the relative FDOM concentration gap (defined as ΔC:C_Surface_) that evaluates the concentration differences between surface water and bottom water along the axial section of the Yangtze Estuary. ΔC is the bottom water concentration (C_Bottom_) subtracting the concentration of surface water (C_Surface_). Clearly, since ΔC:C_Surface_ is below zero, FDOM in surface water was higher than that in bottom water for most stations along the axial section of the Yangtze Estuary in both cruises in 2013. There were higher concentrations of FDOM in bottom water only at station F5 and station E4 in March 2013 and in July 2013, respectively, suggesting DOM sources near the bottom at these two stations.

#### Changes of the ratios between fluorescent components and CDOM along the salinity gradient

**Fig 6:**
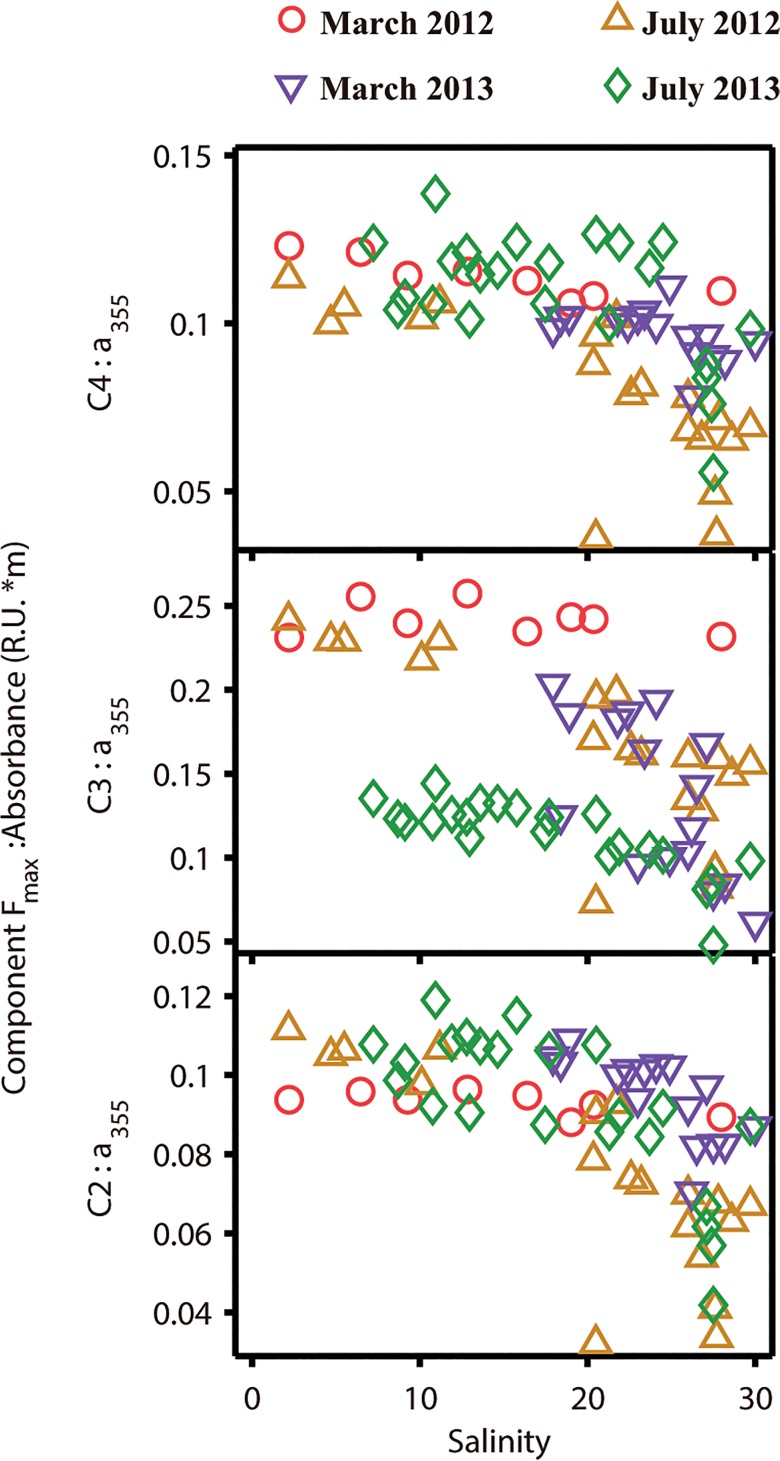
Relationship between salinity and ratios of F_max_ of humic-like components to CDOM absorption coefficient for four cruises.

As mentioned above, the ratio of FDOM to CDOM (F_max_:a_355_) could indicate the photobleaching potential of the DOM, due to the different responses of FDOM and CDOM to sunlight radiation [42]. Fig 6 shows that the F_max_:a_355_ ratios for three humic-like components (C2, C3 and C4) decreased along the salinity gradient during the four sampling periods, although the ratios remained relatively stable in the low to middle salinity ranges. This suggests that photobleaching could be highly dependent on salinity and the turbidity of the studied areas. In addition, the F_max_:a_355_ ratios dropped more significantly in July (∆ data) than in March (O data) for all three humic-like components (C2, C3 and C4) in middle to high salinity region, which could be attributed to the enhanced solar radiation and stronger stratification in July [60, 61].

## Discussion

### Mixing Behavior of FDOM

Different FDOM components showed different mixing behaviors. As reported in previous studies for the Yangtze Estuary [10, 26, 28] as well as some other estuaries [62–65], three humic-like components (C2, C3 and C4) had conservative mixing behavior due to their recalcitrant nature [47, 51]. Meanwhile, the high turbidity and short water retention time [66] resulted in limited photobleaching of humic-like components. Thus, the distribution of humic-like components in the Yangtze Estuary and the surrounding coastal areas was primarily controlled by allochthonous inputs and physical mixing. Unlike humic-like components, the concentrations of two protein-like components (C1 and C5) decreased significantly in low-middle salinity regions (Fig 3), due to the labile nature of these two protein-like components [46, 47] and the enhanced microbial degradation [67].

The conservative mixing behavior (Fig 3 and S1 Table) observed in July 2012 suggests that primary production of phytoplankton exerted no noticeable influences on the FDOM levels or distribution in the Yangtze Estuary [68], since serious algal bloom occurred (Fl. Chl. a was up to 724 μg/L) in the study area during this cruise. Even though FDOM could be produced by microbial conversion of non-FDOM [68], the lag time between primary production by phytoplankton and FDOM production through bacterial conversion may be longer than the study period and the water retention time of the Yangtze Estuary [66]. Therefore, there was no observed increase in FDOM in this study.

### Seasonal and interannual variations of FDOM

The FDOM concentrations in the theoretical freshwater endmember could be indicated by the intercept values derived from linear fitting of FDOM over salinity (S1 Table). For the theoretical freshwater endmember, the FDOM concentration varied between seasons and years. The concentrations of humic-like fluorescent components (C2, C3 and C4) in the theoretical freshwater endmember in 2012 were relatively higher than those in 2013, because there was about 21% more discharge of freshwater in 2012 than in 2013. The high discharge could bring more humic-like materials to the receiving water body [69], resulting in a higher concentration of FDOM in the Yangtze Estuary. Different FDOM components showed different seasonal and interannual patterns in the theoretical freshwater endmember. There were higher concentrations of C2 (0.197 R.U. in 2012 and 0.184 R.U. in 2013) in wet season than those (0.191 R.U. in 2012 and 0.168 R.U. in 2013) observed in dry season. However, there were lower concentrations of C3 (0.426 R.U. in 2012 and 0.224 R.U. in 2013) in high-flow season, compared to those (0.491 R.U. in 2012 and 0.280 R.U. in 2013) in low-flow season. Conversely, C4 showed no clear seasonal but weak interannual pattern (higher concentration in the theoretical freshwater endmember in 2012, compared to 2013). Differences in seasonal and interannual patterns among the three humic-like components suggest both hydrological conditions and FDOM source control the variations of FDOM.

Fig 3 and S1 Table demonstrated some seasonal and interannual variations of FDOM. These variations are caused by freshwater discharge, sources and characteristics of FDOM. For instance, humic-like substances (C2 and C3) may originate from different sources [5, 41, 54], such as precipitation events and pore water release [33, 41], which exhibited seasonal changes. However, the C4 component of FDOM had no apparent seasonal patterns because they are mainly marine humic-like substance or products from microbial activities [36, 47], which showed no seasonal dependence. Likewise, protein-like components, C1 and C5, exhibited no obvious seasonal and interannual patterns, because they are prone to microbial biodegradation [46, 49], and mostly contributed by sewage discharge, salt marshes and pore water release [5, 10, 41].

### Sediment resuspension as the primary DOM source in ETM regions

The DOM concentrations, especially the protein-like components (C1 and C5), were significantly higher in the ETM regions than other regions (Fig 4). This might be related to a series of biogeochemical processes that occur in the ETM region. Due to its shallow depth and tidal forces, strong and frequent sediment resuspension occurred in the ETM region clearly can increase the mixing and levels of FDOM [14, 70], drive the release of DOM-enriched pore water into the overlying water column [33], and therefore increase DOM [10]. In addition, desorption of DOM from POM could contribute to the DOM pool in the ETM region [71]. Meanwhile, photodissolution of POM and flocs has been reported as one important source for DOM in estuary, which could occur in the ETM region [72–74]. Nevertheless, previous studies have showed that FDOM produced through photodissolution was mostly humic-like material [75, 76]. Moreover, enhanced heterotrophic bacterial activity, supported by newly formed flocs in the ETM region, could result in transformation of POM and flocs to DOM and production of DOM [77, 78]. Previous study also showed that products of bacterial transformation and production are most likely to be dissolved amino acids and proteins [79].

The different distribution of fluorescent components in surface and bottom water along the axial section of the Yangtze Estuary (Fig 5) also supports that sediment resuspension was one of the driving force for the DOM release in the ETM region. In most cases, the concentration of DOM (herein FDOM) in surface water is greater than that in bottom water, because the surface water receives more allochthonous input. However, there were higher concentrations of fluorescent components in bottom water than surface water in the stations of F5 and E4 (as indicated by the positive values of ∆C:C_Surface_ in Fig 5). Unlike other stations along F section in March 2013, there was nearly same salinity in surface and bottom water at station F5 (S1 Fig), probably because of the upwelling or vertical mixing that could also induce sediment resuspension [80]. In July 2013, E4 is located at the ETM center and the depth is only 6.5 m. The water could be strongly disturbed by the tidal forces and then sediment resuspension occurs frequently and strongly at this station. At these two stations, released DOM during sediment resuspension firstly reach the overlying bottom water, resulting in higher concentration of DOM in bottom water than in surface water.

There are also other possible allochthonous sources (e.g., tributary input and wetlands) that could contribute DOM (herein FDOM) in the Yangtze Estuary. For example, the input of high-FDOM water from the Huangpu River [41], the last large tributary of Yangtze River, flowing through the metropolitan city of Shanghai, could increase the FDOM level of Yangtze River. However, the highest values of FDOM were observed in ETM regions, instead of the downstream of the Huangpu River outlet (e.g., the station of Y4). Thus, the input from the Huangpu River should not be the primary source for FDOM. Additionally, the large area of wetlands (salt marsh) at the mouth of Yangtze River near the ETM region likely contribute FDOM [5, 81]. However, previous study in the same area showed that salt marsh-derived DOM is dominated by humic-like fluorescence [5], which is not consistent with the protein-dominant DOM as identified in the ETM region in this study. Therefore, tributary input and wetlands output should not be the primary DOM source for the ETM region.

### Photobleaching of DOM

Photobleaching of DOM was minor in the Yangtze Estuary and its adjacent sea, however, it could be monitored combining fluorescence and absorption spectroscopy. The apparently conservative mixing behavior of three humic-like components (C2, C3 and C4) (Fig 3 and S1 Table) suggests photobleaching has a negligible effect on FDOM abundance in the Yangtze Estuary and its adjacent sea, which is consistent with previous studies in the same area [26]. The minor photobleaching has been ascribed to high turbidity and short water retention time in the Yangtze Estuary and adjacent sea. Meanwhile, some scholars have suggested that photobleaching would occur in high-salinity region in the Yangtze Estuary [28, 32]. In this study, the decrease of FDOM:CDOM ratios along the salinity gradient, especially in high-salinity gradient, indicates photobleaching of DOM, lending support to previous guessing. Therefore, photobleaching of DOM could be reflected through changes of DOM quality using fluorescence and absorption spectroscopy.

## Conclusion

Two protein-like components (C1 and C5) and three humic-like components (C2, C3 and C4) were identified from FDOM in the Yangtze Estuary and its adjacent sea. During the four sampling periods in 2012 and 2013, all of the three humic-like fluorescent components showed conservative mixing behavior and were dominated by physical mixing. By contrast, two protein-like components had non-conservative behavior and underwent microbial degradation. Due to hydrological variations, there were higher concentrations of humic-like components in the theoretical freshwater endmember in high-flow year of 2012, compared to the low-flow year of 2013. Meanwhile, there were higher C2 and lower C3 in wet season than in dry season, while there was lack of seasonal patterns for C4 in 2012 and 2013. The results suggest that the composition and transport of DOM from river to ocean would be influenced by the changing climate, especially under extreme drought and flooding. The sediment resuspension in the ETM region is the major mechanism of DOM release, while other processes (e.g., photodissolution of POM, wetland output, tributary input, and microbial production) might contribute to the high concentrations of FDOM (especially the high protein-like components). Future work is needed to quantify the contribution of each DOM source and process in ETM region. The photobleaching removal of humic-like DOM was minor due to high turbidity and short water retention time in the Yangtze Estuary, however, it could be reflected through combination of fluorescence and absorption spectroscopy. In future study, characterization of DOM at molecular level would give more powerful information for tracking changes of DOM during photobleaching.

## Supporting Information

S1 Fig Profile distribution of salinity along the axial section of the Yangtze Estuary in 2013. (EPS)

S1 Table Relationships between three humic-like PARAFAC components and salinity. (DOCX)

## References

[1] Findlay SE, Sinsabaugh RL. Aquatic ecosystems: Interactivity of dissolved organic matter Burlington: Academic Press; 2003.

[2] Benner R, Pakulski JD, Mccarthy M, Hedges JI, Hatcher PG. Bulk Chemical Characteristics of Dissolved Organic Matter in the Ocean. Science. 1992; 255(5051): 1561–1564. 10.1126/science.255.5051.1561 17820170

[3] Cai WJ. Estuarine and coastal ocean carbon paradox: CO2 sinks or sites of terrestrial carbon incineration? Ann Rev Mar Sci. 2011; 3: 123–145. 10.1146/annurev-marine-120709-142723 . 21329201

[4] Bianchi TS. The role of terrestrially derived organic carbon in the coastal ocean: A changing paradigm and the priming effect. Proc Natl Acad Sci USA. 2011; 108(49): 19473–19481. 10.1073/pnas.1017982108 . 22106254 PMC3241778

[5] Gao L, Fan DD, Sun CX, Li DJ, Cai JG. Optical characterization of CDOM in a marsh-influenced environment in the Changjiang (Yangtze River) Estuary. Environ Earth Sci. 2011; 64(3): 643–658. 10.1007/s12665-010-0885-8 .

[6] Gueguen C, Granskog MA, McCullough G, Barber DG. Characterisation of colored dissolved organic matter in Hudson Bay and Hudson Strait using parallel factor analysis. J Mar Syst. 2011; 88(3): 423–433. 10.1016/j.jmarsys.2010.12.001 .

[7] Helms JR, Stubbins A, Ritchie JD, Minor EC, Kieber DJ, Mopper K. Absorption spectral slopes and slope ratios as indicators of molecular weight, source, and photobleaching of chromophoric dissolved organic matter. Limnol Oceanogr. 2008; 53(3): 955–969. 10.4319/lo.2008.53.3.0955 .

[8] Hernes PJ, Benner R. Photochemical and microbial degradation of dissolved lignin phenols: Implications for the fate of terrigenous dissolved organic matter in marine environments. J Geophys Res Oceans. 2003; 108(C9): 3291 10.1029/2002jc001421 .

[9] Rochelle-Newall EJ, Fisher TR. Chromophoric dissolved organic matter and dissolved organic carbon in Chesapeake Bay. Mar Chem. 2002; 77(1): 23–41. 10.1016/S0304-4203(01)00073-1 .

[10] Guo WD, Yang LY, Zhai WD, Chen WZ, Osburn CL, Huang X, et al Runoff‐mediated seasonal oscillation in the dynamics of dissolved organic matter in different branches of a large bifurcated estuary—The Changjiang Estuary. J Geophys Res Biogeo. 2014; 119(5): 776–793. 10.1002/2013JG002540

[11] Alvarez-Salgado XA, Miller AEJ. Dissolved organic carbon in a large macrotidal estuary (the Humber, UK): Behaviour during estuarine mixing. Mar Pollut Bull. 1998; 37(3–7): 216–224. 10.1016/S0025-326X(98)00156-8 .

[12] Bodineau L, Thoumelin G, Béghin V, Wartel M. Particulate organic matter composition in the Estuarine Turbidity Maxima (ETM) of the Seine River estuary. Hydrobiologia. 1998; 373: 281–295. 10.1023/A:1017083924578 .

[13] Huguet A, Vacher L, Relexans S, Saubusse S, Froidefond J-M, Parlanti E. Properties of fluorescent dissolved organic matter in the Gironde Estuary. Org Geochem. 2009; 40(6): 706–719. 10.1016/j.orggeochem.2009.03.002 .

[14] Li JF, Zhang C. Sediment resuspension and implications for turbidity maximum in the Changjiang Estuary. Mar Geol. 1998; 148(3): 117–124. 10.1016/S0025-3227(98)00003-6 .

[15] Mannino A, Harvey HR. Terrigenous dissolved organic matter along an estuarine gradient and its flux to the coastal ocean. Org Geochem. 2000; 31(12): 1611–1625. 10.1016/S0146-6380(00)00099-1 .

[16] Liu JP, Li AC, Xu KH, Velozzi DM, Yang ZS, Milliman JD, et al Sedimentary features of the Yangtze River-derived along-shelf clinoform deposit in the East China Sea. Cont Shelf Res. 2006; 26(17–18): 2141–2156. 10.1016/j.csr.2006.07.013 .

[17] Chai C, Yu ZM, Shen ZL, Song XX, Cao XH, Yao Y. Nutrient characteristics in the Yangtze River Estuary and the adjacent East China Sea before and after impoundment of the Three Gorges Dam. Sci Total Environ. 2009; 407(16): 4687–4695. 10.1016/j.scitotenv.2009.05.011 . 19467558

[18] Tseng YF, Lin J, Dai M, Kao SJ. Joint effect of freshwater plume and coastal upwelling on phytoplankton growth off the Changjiang River. Biogeosciences. 2014; 11(2): 409–423. 10.5194/bg-11-409-2014

[19] Zhang J, Liu SM, Ren JL, Wu Y, Zhang GL. Nutrient gradients from the eutrophic Changjiang (Yangtze River) Estuary to the oligotrophic Kuroshio waters and re-evaluation of budgets for the East China Sea Shelf. Prog Oceanogr. 2007; 74(4): 449–478. 10.1016/j.pocean.2007.04.019 .

[20] Huang QH, Shen HT, Wang ZJ, Liu XC, Fu RB. Influences of natural and anthropogenic processes on the nitrogen and phosphorus fluxes of the Yangtze Estuary, China. Reg Environ Change. 2006; 6(3): 125–131. 10.1007/s10113-005-0001-x .

[21] Liu LS, Zhou J, Zheng BH, Cai WQ, Lin KX, Tang JL. Temporal and spatial distribution of red tide outbreaks in the Yangtze River Estuary and adjacent waters, China. Mar Pollut Bull. 2013; 72(1): 213–221. 10.1016/j.marpolbul.2013.04.002 . 23628547

[22] Romera-Castillo C, Sarmento H, Alvarez-Salgado XA, Gasol JM, Marrase C. Production of chromophoric dissolved organic matter by marine phytoplankton. Limnol Oceanogr. 2010; 55(1): 446–454. 10.4319/lo.2010.55.1.0446 .

[23] Chari NVHK, Keerthi S, Sarma NS, Pandi SR, Chiranjeevulu G, Kiran R, et al Fluorescence and absorption characteristics of dissolved organic matter excreted by phytoplankton species of western Bay of Bengal under axenic laboratory condition. J Exp Mar Biol Ecol. 2013; 445(0): 148–155. 10.1016/j.jembe.2013.03.015

[24] Vodacek A, Blough NV, DeGrandpre MD, Peltzer ET, Nelson RK. Seasonal variation of CDOM and DOC in the Middle Atlantic Bight: Terrestrial inputs and photooxidation. Limnol Oceanogr. 1997; 42(4): 674–686. 10.4319/lo.1997.42.4.0674

[25] Helms JR, Stubbins A, Perdue EM, Green NW, Chen H, Mopper K. Photochemical bleaching of oceanic dissolved organic matter and its effect on absorption spectral slope and fluorescence. Mar Chem. 2013; 155: 81–91. 10.1016/j.marchem.2013.05.015 .

[26] Guo WD, Stedmon CA, Han YC, Wu F, Yu XX, Hu MH. The conservative and non-conservative behavior of chromophoric dissolved organic matter in Chinese estuarine waters. Mar Chem. 2007; 107(3): 357–366. 10.1016/j.marchem.2007.03.006 .

[27] Yang F, Huang QH, Li JH, Zhu XM. Characterization of chromophoric dissolved organic matter in the Yangtze Estuary by absorption and fluorescence spectroscopy. J Environ Sci Sustain Soc. 2007; 1: 55–60.

[28] Sun QY, Wang C, Wang PF, Hou J, Ao YH. Absorption and fluorescence characteristics of chromophoric dissolved organic matter in the Yangtze Estuary. Environ Sci Pollut Res. 2014; 21(5): 3460–3473. 10.1007/s11356-013-2287-4 . 24243263

[29] Köhler H, Meon B, Gordeev V, Spitzy A, Amon R. Dissolved organic matter (DOM) in the estuaries of Ob and Yenisei and the adjacent Kara Sea, Russia. Proc Mar Sci. 2003; 6: 281–310.

[30] Fichot CG, Benner R. The spectral slope coefficient of chromophoric dissolved organic matter (S275-295) as a tracer of terrigenous dissolved organic carbon in river-influenced ocean margins. Limnol Oceanogr. 2012; 57(5): 1453–1466. 10.4319/lo.2012.57.5.1453 .

[31] Shank GC, Evans A. Distribution and photoreactivity of chromophoric dissolved organic matter in northern Gulf of Mexico shelf waters. Cont Shelf Res. 2011; 31(10): 1128–1139. 10.1016/j.csr.2011.04.009 .

[32] Wang Y, Zhang D, Shen ZY, Chen J, Feng CH. Characterization and spacial distribution variability of chromophoric dissolved organic matter (CDOM) in the Yangtze Estuary. Chemosphere. 2014; 95: 353–362. 10.1016/j.chemosphere.2013.09.044 . 24134893

[33] Wang Y, Zhang D, Shen ZY, Feng CH, Chen J. Revealing Sources and Distribution Changes of Dissolved Organic Matter (DOM) in Pore Water of Sediment from the Yangtze Estuary. PLoS ONE. 2013; 8(10): e76633 10.1371/journal.pone.0076633 24155904 PMC3796548

[34] Amado AM, Cotner JB, Cory RM, Edhlund BL, McNeill K. Disentangling the interactions between photochemical and bacterial degradation of dissolved organic matter: amino acids play a central role. Microb Ecol. 2015; 69(3): 554–566. 10.1007/s00248-014-0512-4 . 25351141

[35] Coble PG, Green SA, Blough NV, Gagosian RB. Characterization of dissolved organic matter in the Black Sea by fluorescence spectroscopy. Nature. 1990; 348(6300): 432–435. 10.1038/348432a0

[36] Coble PG. Characterization of marine and terrestrial DOM in seawater using excitation emission matrix spectroscopy. Mar Chem. 1996; 51(4): 325–346. 10.1016/0304-4203(95)00062-3 .

[37] Bro R. PARAFAC. Tutorial and applications. Chemometrics Intellig Lab Syst. 1997; 38(2): 149–171. 10.1016/S0169-7439(97)00032-4 .

[38] Stedmon CA, Markager S, Bro R. Tracing dissolved organic matter in aquatic environments using a new approach to fluorescence spectroscopy. Mar Chem. 2003; 82(3–4): 239–254. 10.1016/S0304-4203(03)00072-0 .

[39] Stedmon CA, Bro R. Characterizing dissolved organic matter fluorescence with parallel factor analysis: a tutorial. Limnol Oceanogr Methods. 2008; 6: 572–579. 10.4319/lom.2008.6.572 .

[40] Kowalczuk P, Durako MJ, Young H, Kahn AE, Cooper WJ, Gonsior M. Characterization of dissolved organic matter fluorescence in the South Atlantic Bight with use of PARAFAC model: Interannual variability. Mar Chem. 2009; 113(3): 182–196. 10.1016/j.marchem.2009.01.015

[41] Dong QQ, Li PH, Huang QH, Abdelhafez AA, Chen L. Occurrence, polarity and bioavailability of dissolved organic matter in the Huangpu River, China. J Environ Sci (China). 2014; 26(9): 1843–1850. 10.1016/j.jes.2014.06.020 . 25193833

[42] Stedmon CA, Nelson NB. The Optical Properties of DOM in the Ocean In: Carlson DAHA, editor. Biogeochemistry of Marine Dissolved Organic Matter (Second Edition). Boston: Academic Press; 2015 p. 481–508.

[43] Murphy KR, Butler KD, Spencer RG, Stedmon CA, Boehme JR, Aiken GR. Measurement of dissolved organic matter fluorescence in aquatic environments: an interlaboratory comparison. Environ Sci Technol. 2010; 44(24): 9405–9412. 10.1021/es102362t . 21069954

[44] Osburn CL, Wigdahl CR, Fritz SC, Saros JE. Dissolved organic matter composition and photoreactivity in prairie lakes of the US Great Plains. Limnol Oceanogr. 2011; 56(6): 2371–2390. 10.4319/lo.2011.56.6.2371

[45] Yamashita Y, Cory RM, Nishioka J, Kuma K, Tanoue E, Jaffé R. Fluorescence characteristics of dissolved organic matter in the deep waters of the Okhotsk Sea and the northwestern North Pacific Ocean. Deep Sea Res Part II Top Stud Oceanogr. 2010; 57(16): 1478–1485. 10.1016/j.dsr2.2010.02.016

[46] Fellman JB, D’Amore DV, Hood E, Boone RD. Fluorescence characteristics and biodegradability of dissolved organic matter in forest and wetland soils from coastal temperate watersheds in southeast Alaska. Biogeochemistry. 2008; 88(2): 169–184. 10.1007/s10533-008-9203-x .

[47] Stedmon CA, Markager S. Tracing the production and degradation of autochthonous fractions of dissolved organic matter by fluorescence analysis. Limnol Oceanogr. 2005; 50(5): 1415–1426. 10.4319/lo.2005.50.5.1415 .

[48] Murphy KR, Stedmon CA, Wenig P, Bro R. OpenFluor- an online spectral library of auto-fluorescence by organic compounds in the environment. Analytical Methods. 2014; 6(3): 658–661. 10.1039/C3ay41935e .

[49] Osburn CL, Del Vecchio R, Boyd TJ. Physicochemical Effects on Dissolved Organic Matter Fluorescence in Natural Waters In: Coble P, Lead J, Baker A, Reynolds D, Spencer RGM, editors. Aquatic Organic Matter Fluorescence. Cambridge: Cambridge University Press; 2014 p. 233–277.

[50] Aiken G. Fluorescence and dissolved organic matter: a chemist's perspective In: Coble P, Lead J, Baker A, Reynolds D, Spencer RGM, editors. Aquatic Organic Matter Fluorescence. Cambridge: Cambridge University Press; 2014 p. 35–74.

[51] Jørgensen L, Stedmon CA, Kragh T, Markager S, Middelboe M, Søndergaard M. Global trends in the fluorescence characteristics and distribution of marine dissolved organic matter. Mar Chem. 2011; 126(1): 139–148. 10.1016/j.marchem.2011.05.002

[52] Yamashita Y, Scinto LJ, Maie N, Jaffé R. Dissolved organic matter characteristics across a subtropical wetland’s landscape: application of optical properties in the assessment of environmental dynamics. Ecosystems. 2010; 13(7): 1006–1019. 10.1007/s10021-010-9370-1

[53] Yamashita Y, Kloeppel BD, Knoepp J, Zausen GL, Jaffé R. Effects of watershed history on dissolved organic matter characteristics in headwater streams. Ecosystems. 2011; 14(7): 1110–1122. 10.1007/s10021-011-9469-z

[54] Coble PG, Spencer RG, Baker A, Reynolds DM. Aquatic Organic Matter Fluorescence In: Coble P, Lead J, Baker A, Reynolds D, Spencer RGM, editors. Aquatic Organic Matter Fluorescence. Cambridge: Cambridge University Press; 2014 p. 75–122.

[55] Helms J, Mao J, Stubbins A, Schmidt-Rohr K, Spencer RM, Hernes P, et al Loss of optical and molecular indicators of terrigenous dissolved organic matter during long-term photobleaching. Aquat Sci. 2014; 76(3): 353–373. 10.1007/s00027-014-0340-0

[56] Cawley KM, Butler KD, Aiken GR, Larsen LG, Huntington TG, McKnight DM. Identifying fluorescent pulp mill effluent in the Gulf of Maine and its watershed. Mar Pollut Bull. 2012; 64(8): 1678–1687. 10.1016/j.marpolbul.2012.05.040 . 22768803

[57] Murphy KR, Bro R, Stedmon CA. Chemometric Analysis of Organic Matter Fluorescence In: Coble P, Lead J, Baker A, Reynolds D, Spencer RGM, editors. Aquatic Organic Matter Fluorescence: Cambridge University Press; 2014 p. 339–375.

[58] Osburn CL, Handsel LT, Mikan MP, Paerl HW, Montgomery MT. Fluorescence tracking of dissolved and particulate organic matter quality in a river-dominated estuary. Environ Sci Technol. 2012; 46(16): 8628–8636. 10.1021/es3007723 22803700

[59] Chen RF, Bada JL. The fluorescence of dissolved organic matter in seawater. Mar Chem. 1992; 37(3): 191–221. 10.1016/0304-4203(92)90078-O

[60] Zhang YL, Liu XH, Osburn CL, Wang MZ, Qin BQ, Zhou YQ. Photobleaching Response of Different Sources of Chromophoric Dissolved Organic Matter Exposed to Natural Solar Radiation Using Absorption and Excitation–Emission Matrix Spectra. PLoS ONE. 2013; 8(10): e77515 10.1371/journal.pone.0077515 24204852 PMC3808427

[61] D'Sa EJ, DiMarco SF. Seasonal variability and controls on chromophoric dissolved organic matter in a large river-dominated coastal margin. Limnol Oceanogr. 2009; 54(6): 2233–2242 10.4319/lo.2009.54.6.2233 .

[62] Yamashita Y, Jaffé R, Male N, Tanoue E. Assessing the dynamics of dissolved organic matter (DOM) in coastal environments by excitation emission matrix fluorescence and parallel factor analysis (EEM-PARAFAC). Limnol Oceanogr. 2008; 53(5): 1900–1908. 10.4319/lo.2008.53.5.1900

[63] Markager S, Stedmon CA, Søndergaard M. Seasonal dynamics and conservative mixing of dissolved organic matter in the temperate eutrophic estuary Horsens Fjord. Estuar Coast Shelf Sci. 2011; 92(3): 376–388. 10.1016/j.ecss.2011.01.014 .

[64] Osburn CL, Stedmon CA. Linking the chemical and optical properties of dissolved organic matter in the Baltic–North Sea transition zone to differentiate three allochthonous inputs. Mar Chem. 2011; 126(1): 281–294. 10.1016/j.marchem.2011.06.007

[65] Guo WD, Yang LY, Hong HS, Stedmon CA, Wang FL, Xu J, et al Assessing the dynamics of chromophoric dissolved organic matter in a subtropical estuary using parallel factor analysis. Mar Chem. 2011; 124(1): 125–133. 10.1016/j.marchem.2011.01.003 .

[66] Rabouille C, Conley DJ, Dai MH, Cai WJ, Chen CTA, Lansard B, et al Comparison of hypoxia among four river-dominated ocean margins: The Changjiang (Yangtze), Mississippi, Pearl, and Rhone rivers. Cont Shelf Res. 2008; 28(12): 1527–1537. 10.1016/j.csr.2008.01.020 .

[67] Jiao NZ, Tang K, Cai HY, Mao YJ. Increasing the microbial carbon sink in the sea by reducing chemical fertilization on the land. Nat Rev Microbiol. 2011; 9(1): 75–75. 10.1038/nrmicro2386-c2 21113183

[68] Rochelle-Newall EJ, Fisher TR. Production of chromophoric dissolved organic matter fluorescence in marine and estuarine environments: an investigation into the role of phytoplankton. Mar Chem. 2002; 77(1): 7–21. 10.1016/S0304-4203(01)00072-X .

[69] Zhang YL, Yin Y, Liu XH, Shi ZQ, Feng LQ, Liu ML, et al Spatial-seasonal dynamics of chromophoric dissolved organic matter in Lake Taihu, a large eutrophic, shallow lake in China. Org Geochem. 2011; 42(5): 510–519. 10.1016/j.orggeochem.2011.03.007 .

[70] Komada T, Schofield OM, Reimers CE. Fluorescence characteristics of organic matter released from coastal sediments during resuspension. Mar Chem. 2002; 79(2): 81–97. 10.1016/S0304-4203(02)00056-7

[71] Yang LY, Guo WD, Hong HS, Wang GZ. Non-conservative behaviors of chromophoric dissolved organic matter in a turbid estuary: Roles of multiple biogeochemical processes. Estuar Coast Shelf Sci. 2013; 133: 285–292. 10.1016/j.ecss.2013.09.007 .

[72] Kieber RJ, Whitehead RF, Skrabal SA. Photochemical production of dissolved organic carbon from resuspended sediments. Limnol Oceanogr. 2006; 51(5): 2187–2195. 10.4319/lo.2006.51.5.2187 .

[73] Mayer LM, Schick LL, Skorko K, Boss E. Photodissolution of particulate organic matter from sediments. Limnol Oceanogr. 2006; 51(2): 1064–1071. .

[74] Helms JR, Glinski DA, Mead RN, Southwell MW, Avery GB, Kieber RJ, et al Photochemical dissolution of organic matter from resuspended sediments: Impact of source and diagenetic state on photorelease. Org Geochem. 2014; 73: 83–89. 10.1016/j.orggeochem.2014.05.011 .

[75] Liu Q, Shank GC. Solar Radiation-Enhanced Dissolution (Photodissolution) of Particulate Organic Matter in Texas Estuaries. Estuaries and Coasts. 2015: 1–13. 10.1007/s12237-014-9932-0

[76] Pisani O, Yamashita Y, Jaffé R. Photo-dissolution of flocculent, detrital material in aquatic environments: Contributions to the dissolved organic matter pool. Water Res. 2011; 45(13): 3836–3844. 10.1016/j.watres.2011.04.035 21570101

[77] Asmala E, Bowers DG, Autio R, Kaartokallio H, Thomas DN. Qualitative changes of riverine dissolved organic matter at low salinities due to flocculation. J Geophys Res Biogeo. 2014; 119(10): 1919–1933. 10.1002/2014jg002722 .

[78] Cammack WKL, Kalff J, Prairie YT, Smith EM. Fluorescent dissolved organic matter in lakes: Relationships with heterotrophic metabolism. Limnol Oceanogr. 2004; 49(6): 2034–2045. 10.4319/lo.2004.49.6.2034 .

[79] Rosenstock B, Simon M. Sources and sinks of dissolved free amino acids and protein in a large and deep mesotrophic lake. Limnol Oceanogr. 2001; 46(3): 644–654. 10.4319/lo.2001.46.3.0644

[80] Dale AW, Prego R. Physico-biogeochemical controls on benthic-pelagic coupling of nutrient fluxes and recycling in a coastal upwelling system. Mar Ecol Prog Ser. 2002; 235: 15–28. 10.3354/meps235015 .

[81] Clark CD, Litz LP, Grant SB. Saltmarshes as a source of chromophoric dissolved organic matter (CDOM) to Southern California coastal waters. Limnol Oceanogr. 2008; 53(5): 1923–1933. 10.4319/lo.2008.53.5.1923

