# Supplementary material for: Spatiotemporal Distribution, Sources, and Photobleaching Imprint of Dissolved Organic Matter in the Yangtze Estuary and Its Adjacent Sea Using Fluorescence and Parallel Factor Analysis
Source: PLoS One. 2015 Jun 24;10(6):e0130852. doi: 10.1371/journal.pone.0130852 (PMC4479555; doi:10.1371/journal.pone.0130852)
Supplement: S1 Table — (DOCX) [file pone.0130852.s002.docx]

**S1 Table Relationships between three humic-like PARAFAC components and salinity.**

| Parameters (R.U.) | | March 2012  (dry season, n=36) | July 2012  (wet season, n=35) | March 2013  (dry season, n=52) | July 2013  (wet season, n=23) | Fitting results for four sampling periods (n=146) |
| --- | --- | --- | --- | --- | --- | --- |
| C2 | intercept | 0.191±0.004 | 0.197±0.006 | 0.168±0.005 | 0.184±0.009 | 0.185±0.003 |
|  | slope | -0.0051±0.0001 | -0.0056±0.0002 | -0.0047±0.0002 | -0.0052±0.0005 | -0.0051±0.0001 |
|  | r | 0.989 | 0.972 | 0.968 | 0.923 | 0.970 |
|  | r^2^ | 0.977 | 0.945 | 0.938 | 0.853 | 0.941 |
|  | *p* value | <0.001 | <0.001 | <0.001 | <0.001 | <0.001 |
| C3 | intercept | 0.491±0.011 | 0.426±0.014 | 0.280±0.015 | 0.224±0.012 | 0.337±0.013 |
|  | slope | -0.0129±0.0004 | -0.0119±0.0005 | -0.0080±0.0005 | -0.0064±0.0006 | -0.0093±0.0005 |
|  | r | 0.987 | 0.972 | 0.921 | 0.914 | 0.859 |
|  | r^2^ | 0.974 | 0.944 | 0.849 | 0.835 | 0.739 |
|  | *p* value | <0.001 | <0.001 | <0.001 | <0.001 | <0.001 |
| C4 | intercept | 0.236±0.006 | 0.199±0.006 | 0.161±0.005 | 0.197±0.011 | 0.202±0.004 |
|  | slope | -0.0062±0.0002 | -0.0056±0.0002 | -0.0043±0.0002 | -0.0052±0.0006 | -0.0055±0.0001 |
|  | r | 0.982 | 0.977 | 0.962 | 0.887 | 0.953 |
|  | r^2^ | 0.963 | 0.954 | 0.925 | 0.787 | 0.908 |
|  | *p* value | <0.001 | <0.001 | <0.001 | <0.001 | <0.001 |
